# Between the city and the farm: food environments in artisanal mining communities in Upper Guinea

**DOI:** 10.1017/S1368980021002020

**Published:** 2022-02

**Authors:** Stella Nordhagen, Mohamed L Fofana, Alpha O Barry, Sadio Diallo, Joseph L Songbono, Ronald Stokes-Walters, Laetitia X Zhang, Rolf Klemm, Peter J Winch

**Affiliations:** 1 Global Alliance for Improved Nutrition (GAIN), Rue Varembé 7, Geneva 1202, Switzerland; 2 Helen Keller International, Washington, DC, USA; 3 Helen Keller International Guinea, Landreah, Dixinn Corniche, Conakry, Guinea; 4 Julius Nyerere University of Kankan, Kankan, Guinea; 5 Department of International Health, Johns Hopkins Bloomberg School of Public Health, Baltimore, MD, USA; 6 Action Against Hunger USA, New York, NY, USA

**Keywords:** Artisanal mining, Food environment, Food choice, Rural households, Guinea, West Africa

## Abstract

**Objective:**

Artisanal and small-scale mining (ASM) is a widespread livelihood in low- and middle-income countries; however, many in ASM communities face high levels of poverty and malnutrition. The food environments in ASM communities have non-agricultural rural characteristics that differ from those in urban and agricultural rural areas examined in much existing food environment literature.

**Design:**

We examine these complex external and personal food environments in ASM communities via a study using qualitative and quantitative methods. Market surveys and a cross-sectional household survey, plus qualitative mining site non-participant observations and in-depth structured interviews, were conducted in three waves.

**Setting:**

Eighteen study sites in ASM communities in northern Guinea.

**Participants:**

Surveys covered mothers in mining households with young children (*n* 613); in-depth interviews engaged caregivers of young children (*n* 45), food vendors (*n* 40) and young single miners (*n* 15); observations focused on mothers of young children (*n* 25).

**Results:**

The external food environment in these ASM communities combines widespread availability of commercially processed and staple-heavy foods with lower availability and higher prices for more nutritious, non-staple foods. Within the personal food environment, miners are constrained in their food choices by considerable variability in daily cash income and limited time for acquisition and preparation.

**Conclusions:**

We demonstrate that ASM communities have characteristics of both urban and rural populations and argue for greater nuance and appreciation of complexity in food environment research and resultant policy and programming.

There is increasing recognition of the food environment’s role in shaping food choices^(1–5)^. It is within the food environment that people obtain and consume foods, and food environment interventions can potentially have larger reach than attempts to change personal behaviours.^(5)^ Despite the importance of the food environment for consumers everywhere, most food environment research has examined food environments in high-income countries^(6)^. However, populations in low- and middle-income countries (LMIC) face large burdens of undernutrition as well as growing prevalence of overweight/obesity^(7)^, with potentially serious negative consequences for health and well-being^(8)^.

Examining food environments in LMIC, researchers commonly examine one of two scenarios: urban populations^(9,10)^ or rural subsistence-agricultural households^(11,12)^. For the former, food choice is shaped by cash income and market food availability and prices – i.e. the external food environment, which is recognised to be dynamic and complex in urban areas, and sometimes associated with urbanisation itself^(13–15)^. In the latter, food choice is shaped by the household’s own production, ability to sell/store surplus production and purchase of additional food with agricultural income – normally raw ingredients, as opposed to prepared foods.

The changing nature of food systems is blurring the divisions between ‘urban’ and ‘rural’ food environments and ‘traditional’ and ‘modern’ food systems^(16)^. Food now travels through longer supply chains, spanning rural and urban areas, with greater levels of processing, packaging and marketing^(16–18)^. Markets are important sources of food for rural populations^(18–21)^, and agriculture in urban areas is widespread across many African cities^(22)^. Both undernutrition and overweight/obesity now span urban and rural zones^(16)^, with overweight/obesity levels rising fastest in rural areas^(23)^. These overlapping characteristics suggest increasing complexity and fluidity of rural food environments. However, rural areas where non-agricultural livelihoods are common have rarely been considered in food environment research; by considering only certain aspects of typical rural and urban areas, researchers may not have fully elucidated those qualities relevant to both.

In this paper, we delve into this complexity through a study of artisanal mining communities in Guinea – areas fusing rich mineral resources with widespread poverty and malnutrition. Drawing on data from a multi-phase study, we demonstrate that these communities blend urban and rural characteristics, with complex food environments that cannot be easily categorised within the typologies described by prior research^(24)^. This paper adds to a small literature on food environments in LMIC^(6)^, bringing methodological diversity and a new focus on non-agricultural rural settings. It also adds to the literature on socio-economic aspects and consequences of resource extraction livelihoods^(25–29)^ and can inform discussions of nutrition changes during structural transformation of rural areas, in which households tend to allocate more time to off-farm labor, such as mining^(30)^.

## Food environments and food choice

Building on earlier conceptual work^(20,31–33)^, Turner et al. define the food environment as, ‘the interface that mediates people’s food acquisition and consumption within the wider food system (encompassing) external dimensions such as availability, prices, vendor and product properties, and promotional information; and personal dimensions such as the accessibility, affordability, convenience and desirability of food sources and products^(34)^.’ The external dimensions reflect absolute characteristics applicable to all, whereas the internal dimensions are specific to the individual. For example, *availability* refers to a food’s presence; for individuals, this translates into *accessibility* based on time availability and mobility. Similarly, prices translate into affordability, vendor/product properties map onto convenience and marketing/regulation affect desirability^(34)^.

Empirical work on food environments has focused on high-income countries and, within those, primarily on urban areas and issues related to obesity/overweight^(35–38)^. Work in LMIC has been limited, despite numerous differences with high-income countries – such as more informal retailers, greater price volatility, less advertising/labelling and limited regulation. A recent review of food environment research in LMIC found that no articles examined low-income countries and most (70 %) focused on upper-middle-income countries^(6)^. Only half of studies considered both external and personal food environments, and only 11 % used mixed methods. Moreover, only five of 70 studies focused on specific rural areas^(11,39–42)^. Of those, four focused on Latin America; two considered agricultural communities, while the remaining three offered no details on livelihoods. A recent categorisation of food environments also largely omitted non-agrarian rural settings^(24)^. There is thus a gap in research on food environments in rural LMIC, particularly for rural non-agricultural households and low-income African countries.

## Artisanal and small-scale mining

Artisanal and small-scale mining (ASM) is a growing livelihood supporting approximately 100 million people in eighty LMIC, typically practiced by poor, under-educated populations in rural areas^(43)^. Artisanal miners generally work independently and informally, with rudimentary techniques, poor conditions and low returns^(44)^. In Guinea, ASM is estimated to engage 222 000–300 000 people and financially support about 1·3–1·5 million – 12 % of the population^(26,45)^. In the country’s northeast, mining (particularly of gold) is a major economic activity. Men, women and children all participate^(46–48)^, with women estimated to comprise up to 74 % of small-scale miners in Guinea, usually in less well-paid roles^(47,49)^.

While ASM can be a route to reduced poverty/vulnerability^(49–51)^, many enter the sector because of hardship; most are poor former or concurrent subsistence farmers^(26)^. ASM is associated with numerous environmental and health risks, including malnutrition. The region studied here has stunting levels among children under 5 years (30·5 %) on par with the national average but the country’s second-highest prevalence of wasting among under-fives (10·7 %); among women (ages 15–49 years), 6·7 % are underweight and 24·6 % are overweight/obese, based on standard BMI cutoffs^(52)^. Nutritious food may be hard to access due to high costs, limited options for producing/purchasing, seasonality and time constraints. Mining sites may become host to temporary shanty towns, with scant resources and damaged soils or water unsuitable for agriculture^(44,53,54)^. Fresh food access may be further limited if agriculture is displaced by mining^(26,55,56)^.

Given certain characteristics associated with urban populations – e.g. access to cash, purchasing most food – ASM areas exemplify food environments mixing urban and rural characteristics. This agriculture-to-mining transition is also representative of larger shifts from locally focused agricultural livelihoods to (peri-)urban living and unstable livelihoods dependent on global markets^(57,58)^, with concomitant shifts in food environments, diets and nutrition^(24,59)^.

There has been little study of food and nutrition within ASM communities. A spatial modelling study in Democratic Republic of Congo found high malnutrition rates in provinces reliant on mining^(60)^. An analysis of data from thirty-eight African countries showed that internationally operated industrial mines are associated with decreased food availability among women (but not men) and lower children’s dietary diversity but did not consider ASM^(61)^. Researching Guinean bauxite miners, Dabo reported that women saw mining as causing maternal and child malnutrition^(53)^. Examining ASM in Mali, Keita noted that, amid a cereal-based diet, high consumption of low-quality canned foods, unhygienic conditions and intensive energy expenditure, malnutrition was prevalent^(62)^. Tschakert mentioned that Ghana’s artisanal gold miners saw poor quality and limited food as problems with mining^(63)^. In contrast, Grätz cited miners’ excessive food consumption in boom times in Benin^(25)^. Finally, Maconachie and collaborators examined connections between agriculture and ASM in Sierra^(28,51,64–66)^. None of these authors, however, examined nutrition or food environments further.

## Methods

Research was conducted in Kouroussa and Siguiri prefectures, Kankan region, Guinea. Study sites were selected purposively to cover four different strata: temporary mining camps, villages far from towns, villages close to towns and towns. Stratification was driven by expectations that proximity to a village/town influenced food access. Eighteen sites were included; specific sites studied changed over time, as mines are regularly closing and being started.

The focus population was mothers of children under age 5 in mining households due to the importance of maternal and young child nutrition in development. Young single miners, primarily male, were also included to understand how experiences might vary across groups. Data were collected iteratively from May 2018 to December 2019. First, structured non-participant observations at ten mining sites were used to understand the setting, food availability, actors involved and work roles. Stakeholders (government, civil society and local ASM leaders) were consulted via key informant interviews. A cross-sectional household survey was carried out in two waves, with no overlap in respondents between waves. Households were chosen randomly, with sample size proportional to site population. Survey sample size was determined based on an ability to calculate the proportion of women with a minimally diverse diet, conservatively assuming a level of 50 %, alpha of 0·05, power of 0·8, beta of 0·2 and design effect of 1·5, yielding a minimum sample size of 291 per wave. Households were eligible to participate if at least one member was active in mining and a child under 5 lived in the household. The mother/guardian of the youngest child was the primary respondent. In addition, two waves of in-depth interviews were conducted, covering mothers of young children, food vendors and young single miners. Twenty-five interviews with mothers also included extended periods of non-participant observation. Four market surveys were conducted, aiming to cover all major seasons. In each, 4–7 markets that served the mining sites targeted by the household survey were selected; the number of markets surveyed varied by wave, depending on how many sites had associated markets. In-depth non-participant observations were carried out at eight markets.

Data collection instruments were developed based on standard questions (e.g. from Demographic and Health Surveys) and validated tools used by other studies within the Drivers of Food Choice project portfolio*,* where relevant. Instruments were pilot-tested in a comparable community not targeted by the research and revised accordingly, primarily to shorten interview length. The survey and interview questionnaires were developed in French and translated orally into Malinké; an agreed-upon translation was developed and validated with interviewers during training. Household and market surveys used structured, mainly closed-ended questionnaires. Market surveys collected price and quantity data and covered all foods at least occasionally present in local markets aside from the ‘basic ingredients and snacks’ categories (see Table 5), wherein there was more diversity and indicative products were chosen to represent broader groups. Household survey questions on food acquisition measured the more frequently consumed foods (Table 6). Commercially processed drinks and snacks (e.g. cookies, candies, pasta and sardines), following a definition previously used for research in Guinea^(67)^, were also noted in observations. For the household and market observations, semi-structured paper-based guides were used, including both categorical/closed-ended questions (e.g. distance to closest village) and open-ended ones (e.g. miners’ working conditions).

Field research was conducted by up to nine data collectors, supervised by 2–5 supervisors (depending on the data collection wave). Data collectors were competitively selected from recent graduates of Université Julius Nyerere de Kankan’s sociology department. All knew the local context well and were fluent in Malinké and French. Data collectors were trained in research ethics, interview methods and data-entry and note-taking methods in addition to the data-collection instruments. Each data collector specialised in either qualitative (in-depth interviews, observations) or quantitative (household and market survey) methods. Supervisors oversaw data collection progress and verified data quality daily.

Data from market and household surveys were entered via smartphones into a cloud-based data storage platform. Interview data were recorded via detailed interview notes and post-interview debriefings. During the non-participant observations, notes were taken on paper and later scanned/transcribed into electronic versions. All data were entered and analysed in French.

Interview notes were coded using deductive coding in ATLAS.ti^(68)^. An initial deductive coding scheme was created based on the thematic modules covered in the interview guides and applied to all collected data; similar codes were grouped into thematic categories (e.g. food preparation practices) and code summaries created. Quantitative data were cleaned and analysed using Stata SE15^(69)^ via frequency tabulations and/or calculation of means. Dietary diversity was assessed via a list-based 24-h-recall using ten standard categories^(70)^. We also calculated the Household Food Insecurity Access Score^(71)^. For food price data, there was considerable variation across seasons and markets; as such, we calculated median price across all data points and the seasonal range in the across-market median price, to indicate seasonal price variability.

Table 1 summarises data collection methods, sample sizes and topics, while Table 2 indicates how these were used to measure different food environment dimensions, showing the diversity in evidence brought by the mix of methods used.

**Table 1:** Summary of methods

Method	Target population	Sample size	Topics
Household surveys	Mothers or guardians of children under the age of five in households engaged in mining	613 (in two waves of ˜300, August 2018, March/April 2019)	Demographics; income & livelihoods; diets; food insecurity; food access, acquisition and preferences; household decision-making
In-depth semi-structured interviews	Mothers of children under age five in mining households	45 (in two waves)	Livelihoods; food access, acquisition, and preferences; household decision-making; childcare
	Vendors (mobile & fixed, including small kiosks/shacks & tables) of food (meals, drinks, & snacks) at mining sites	40 (in two waves)	Livelihoods; food access, acquisition and preferences; vendor decision-making
	Single miners (predominantly male, one female)	15	Livelihoods; food access, acquisition and preferences; household decision-making
non-participant observation	Mothers of children under the age of five in households engaged in mining	25	Food acquisition and preparation practices; child feeding
Market surveys	Public food markets	4–7 markets, at four points in time (23 total data points)	Food availability, prices (assessed for the most basic quality product for each product; per kilogram where feasible and using local units and a conversion factor where not)
Market observations	Public food markets	8	Market organisational structure; physical features; food presence

**Table 2:** Methods and main indicators used by food environment dimension

Food environment dimension	Data collection methods	Main indicators/aspects
External		
Availability	Market surveys (O); household survey (food production & sourcing, R)	Presence of food source; presence of food category or specific food
Prices	Market surveys (O); vendor interviews (R)	Median price; range in median prices; vendors’ pricing strategies
Vendor & Product properties	Market/site observations (O); vendor and consumers interview (P, R)	Vendor demographics; type of vendor (i.e. kiosk, open table); market days; food category or specific food sold; perceptions of food quality and safety
Marketing*	Market/site observations (O); in-depth interview vendors (P)	Presence of advertising; use of promotions
Personal		
Accessibility	Household survey questions (food production & sourcing, food store access, P/R); consumers interviews (perceptions of accessibility, P)	Walking time to food sources; share of food sourced from different sources (including own production); qualitative perceptions of accessibility
Affordability	Household survey (income, R); consumers interviews (perceptions of relative food affordability, purchasing choices in response to questions about using a hypothetical budget, P)	Self-reported income for an ‘average’ and ‘good’ week; perceptions of relative food affordability; change in stated purchasing choices as hypothetical budget varied
Convenience	Household survey (rating question on key criteria when choosing food, P); vendor and consumer interviews (P); observations (of time use, O)	Importance of convenience-related attributes; vendors’ perceptions of consumers’ convenience preferences; consumers’ perceptions of convenience; time use related to preparing food and eating
Desirability	Household survey (rating question on key criteria when choosing food, P); vendor and consumer interviews (P)	Importance of desirability-related attributes; vendors’ perceptions of consumers’ desirability preferences; consumers’ perceptions of desirability; respondents’ general views on food culture and habits

Dimensions are classified as per^(39)^. To clarify the type of evidence presented on each food environment dimension, we note whether data are objective (via direct observation) (O); objective fact as reported by participants (R) or participants’ personal perceptions (P).

* Initial key informant interviews indicated that regulation was essentially absent from the sites, which was confirmed via initial observations. As such, regulation was not examined in depth.

## Results

### Sample overview and diets

Table 3 presents the summary statistics from the survey respondents; they are also roughly representative of mothers included in in-depth interviews. Food vendors interviewed tended to be women (97·5 %) with children aged 20–55 years. Single miners interviewed were predominantly male (93 %) aged 18–33 years. Most vendors and single miners had migrated from elsewhere in the sub-national region.

**Table 3:** Characteristics of survey respondents

	Frequency	Percent
Respondent characteristics (*n* 613)		
Age		
Mean	26·1	
sd	6·6	
Married (monogamous)	386	46·7 %
Married (polygamous)	312	50·9 %
Illiterate	555	90·5 %
Fully literate	35	5·7 %
Received any education	108	17·6 %
Finished primary school	64	10·4 %
Originally from village/town(s) associated with mine	241	39·3 %
Non-Guinean	6	1·0 %
Main economic activity		
Mining	478	78·0 %
Food selling	42	6·9 %
Other	93	15·2 %
Seasonal inhabitant	115	18·8 %
Muslim	596	97·6 %
Malinké speaking	575	94·1 %
Household characteristics		
Household size		
Mean	8·1	
sd	6·24	
Number of children aged under 5 years in household		
Mean	2·5	
sd	2·7	
Number of children aged 5–15 years in household		
Mean	2·1	
sd	1·2	
Improved toilet	172	28·1 %
Improved water source	293	48·0 %
Food access from own production		
Cultivated agricultural land in past 2 years	366	59·9 %
Has a food garden	99	16·2 %
Owns chickens	288	47·1 %
Owns other livestock	235	38·5 %

Table 4 summarises women’s diets, based on the household survey. Diets are largely inadequate from a nutritional perspective, with only a minority of women meeting the dietary diversity threshold^(70)^. Food groups that are particularly under consumed include egg, dairy, legumes, fruit and green leafy vegetables. Overall, 24·5 % of households are moderately food insecure and 27·6 % are severely food insecure^(71)^.

**Table 4:** Women’s dietary diversity and consumption of major food groups

	Frequency	Percent
Women’s dietary diversity score*		
Mean	3·3	
sd	1·67	
Percent meeting minimum dietary diversity (5 of 10 groups)	140	22·8 %
Percent consuming in prior 24 h…		
Starches and grains	588	96·2 %
Dark green leafy vegetables	155	25·4 %
Other vitamin-A rich fruit and vegetables	250	40·9 %
Other vegetables	304	49·8 %
Other fruit	54	8·8 %
Egg	64	10·5 %
Meat	186	30·4 %
Fish or shellfish	339	55·5 %
Legumes	89	14·6 %
Dairy	87	14·2 %
Sweets	147	24·0 %
Energy drinks†	90	14·7 %
*n*.	611	


* Due to a survey error, the 'pulses’ and 'nuts/seeds’ groups of the standard ‘Minimum Dietary Diversity-Women’ (MMD-W) score were not separated for the first wave of the survey (approximately half of responses). The 'pulses/nuts/seeds’ group formed by combining 'pulses’ and 'nuts/seeds’ using wave 2 data was reported as consumed significantly more often than the combined group in wave 1, implying that MDD-W was underestimated in wave 1 data.

† While not a standard category, energy drinks were included due to initial observations and key informants indicating these as widely consumed.

### External food environment

Key characteristics of the external food environment include food availability, prices, vendor properties and food properties^(34)^. Table 5 offers an overview of the foods available in open-air markets in the zones studied as well as median price and range in median price across seasons, based on market survey data. Basic ingredients (e.g. rice, onions, oil, sugar and salt) are available across all markets, as are some more nutritious foods (e.g. fish, egg). However, several types of vegetables, fruits and animal-source foods cannot be found in all markets. Moreover, prices are high for a number of nutritious options (e.g. beef, chicken) and relatively higher for more nutrient-dense options compared with alternatives (e.g. white rice or maize flour is cheaper than the protein-rich local grain fonio, beans or cowpeas). There is widespread availability of commercially processed, packaged products (e.g. pasta, sardines, cookies and candies). Particularly striking is the number of energy drinks (local or off-brand versions of Red Bull) on offer. Comparing availability and prices across markets associated with different site types (data not shown) suggests that remote mining camps have both the lowest availability and highest prices for many foods. Lower availability was confirmed by an eight-market case study associated with this study^(72)^. Most miners and food vendors opined in interviews that prices at mining sites were often higher than in towns.

**Table 5:** Availability and prices of foods in open-air markets*,†

	Avg. Pct of Markets Selling	Median Price (GNF)	IQR (GNF)	Seasonal range in median price (GNF)		Avg. Pct of Markets Selling	Median Price (GNF)	IQR (GNF)	Seasonal range in median price (GNF)
Grains & Staples					Legumes				
Maize flour	85 %	5000	2000–7666	5000–7000	Cowpea	35 %	7000	6500–7500	7000–10 000
Maize kernels	74 %	5000	2500–6000	3750–5000	Bean	75 %	8500	8000–10 000	7000–10 000
Rice	100 %	6500	6000–7000	6500–6800	Animal-source foods				
Millet	19 %	6000	6000–10 000	6000–10 000	Fresh fish	100 %	25 000	20 000–25 000	22 500–25 000
Sorghum	50 %	7000	6250–7000	6000–7000	Dried fish	92 %	27 000	20 000–40 000	27 000–65 000
Fonio	88 %	10 000	9000–10 000	9500–10 000	Sardines (1 tin)	96 %	5000	5000–5000	–
Wheat flour	57 %	7000	7000–7000	7000–7000	Beef	92 %	30 000	30 000–30 000	30 000–31 250
Pasta/Noodles	100 %	5000	5000–7000	3500–11 000	Local chicken (1 bird)	57 %	47 500	40 000–81 666	40 000–66 250
Bread (1 baguette)	100 %	2000	2000–2000	2000–2000	Imported chicken (1 bird)	96 %	25 000	20 000–25 000	20 000–25 000
Cassava	33 %	3500	2000–5000	2500–5000	Egg (1)	100 %	1500	1500–1875	1500–2000
Plantain	52 %	10 000	10 000–11 000	8000–11 500	Lait caillé (1 l.)	60 %	11 000	10 000–15 000	8000–13 000
White Sweet Potato	59 %	5000	5000–10 000	3333–10 000	Powdered milk (20 g packet)	100 %	1000	1000–1000	–
Orange Sweet Potato	36 %	1750	1000–2000	1000–2000	Basic ingredients				
Potato	80 %	10 000	10 000–12 500	8000–12 500	Oil (1 l.)	100 %	13 750	12 750–15 000	13 000–14 500
Vegetables					Red palm oil (1 l.)	100 %	11 000	10 000–12 000	11 000–12 000
Onion	100 %	10 000	8750–12 000	7000–11 500	Sugar (100 g)	100 %	1000	900–1000	800–1000
Tomato	76 %	1750	1000–3000	2000–4500	Snacks				
Dark leafy greens	89 %	1250	500–2000	1500–4583	Peanuts (small bag)	88 %	800	500–1000	500–950
Eggplant	100 %	1750	1000–3500	2000–8333	Fizzy drink (1 small bottle)	100 %	6500	6000–7000	6000–7000
African Eggplant	100 %	1000	1000–1500	1000–6071	Homemade juice (1 small bottle)	94 %	2000	1500–2000	1000–2000
Lettuce	44 %	5000	3000–5000	5000–9167	Beignets (1)	75 %	750	500–1000	750–1000
Cucumber	31 %	2125	1250–3000	2125–3000	Cake/cupcake (1)	83 %	500	500–1000	500–750
Fruits					Cookies (homemade) (1 pack)	49 %	1000	500–1000	5000–1000
Banana (1, medium)	80 %	1000	1000–1670	813–1666	Cookies (commercial) (1 pack)	100 %	1000	1000–3000	1000–5000
Mango (1, medium)	25 %	2000	1000–2750	1875–2000	Candies (1)	100 %	500	500–500	500–500
Avocado (1, medium)	38 %	3500	2250–5000	2500–5000					
Orange (1, medium)	69 %	1000	500–1000	500–1350					

Avg, average.

* Data are from December 2018 and March, July and December 2019. Price is per kilogram unless otherwise specified, in Guinean Franc (GNF). 10 000 GNF is approximately equal to 1 USD. Unavailable foods (mutton and goat meat) are not included; data for yam and salt are excluded due to inconsistent units across surveys.

† Data are from December 2018 and March, July and December 2019. Medians are reported rather than means due to the presence of outliers in the data. The column ‘seasonal range in median price’ presents the range across seasons of the median price across markets (i.e. one median price was calculated for each of December 2018, March, July and December 2019, and the range in those four median prices is shown here). Price is per kilogram unless otherwise specified, in Guinean Franc (GNF). Over this time period, the GNF was relatively stable, at 9100–9500 GNF to 1 USD. Unavailable foods (mutton and goat meat) are not included; data for yam and salt are excluded due to inconsistent units across surveys.

Qualitative interview responses offered insights into how ASM impacts food availability. For example, a 21-year-old female miner noted, ‘It’s hard to find food to eat here, but in town, it’s easy; the difference is that here the vendors don’t give enough food… in town, all types of food are available….’ One vendor stated bluntly, ‘The clients (at the mining site) are required to buy what they find from us, even if the price is expensive’ (45-year-old female). Some vendors also mine, which further steers them to offering easy, convenient foods: ‘I sell (commercially processed foods) because they don’t take much care to maintain, that gives me time to go mine myself, then come back and sell them’ (male snack vendor, age unknown).

Observations showed that available food outlets in villages serving mining sites include open-air markets (daily or weekly) and small shops/kiosks selling mainly packaged foods as well as basic prepared foods (e.g. omelets). Mining sites themselves have few unprepared foods or ingredients for sale but host numerous mobile vendors of prepared foods as well as basic cafes. Survey data indicate that, from their home, 88 % of respondents have a prepared food vendor within a 15-minute walk, whereas only 48 % have access to a large store and 60 % to an open-air market (which may not be open daily). Food vendors are generally women and come from other areas; interview responses suggested most to be attracted by the lucrative opportunity offered by mining sites: ‘I don’t know anything about mining,’ one vendor noted, ‘I just come here to sell (my food) … I just sell food because that’s where I have luck, that’s where I earn money’ (34-year-old female).

Many vendors explained that they integrate preparation of food to sell into cooking for their families. Some sell from a bowl on the ground; others set up temporary stalls with tables. They mainly offer cooked meals aligning to local recipes (e.g. rice with sauce); some also offer drinks (e.g. water in small bags). Service is fast, and they locate themselves close to mine shafts to allow miners to minimise time spent not working. Cafes are more likely to be run by men and offer commercially processed drinks (e.g. instant coffee and energy drinks) and basic foods (e.g. omelets and sandwiches).

Observations and interviews showed that foods on sale at sites include traditional dishes aligning to local preferences as well as commercially processed foods once only available in cities. All foods are ready-to-eat and available quickly; most are either cheap (e.g. individually sold candies) or sold in a range of sizes at different prices. For example, the price of a plate of rice depends on the portion size and how many meat pieces are included. While nutritious options exist, observations and interviews suggest the lowest-priced meal options are heavily dependent on staple grains (e.g. rice with a bit of thin sauce). Most snack options are high on refined sugars and low on micronutrients or protein (e.g. packaged cookies). All commercially processed foods and some homemade ones come in plastic packaging for protection from the mine’s dust. However, several interviewees mentioned that poor-quality (e.g. spoiled, expired, or damaged) food was commonly sold. As one food vendor noted, ‘While some (commercially processed foods) exist in Conakry, too… the food quality…that exists in Conakry does not exist here’ (52-year-old female). Poor quality and limited availability were attributed to poor infrastructure, including poor-quality roads and little electricity or running water.

We found that sites typically hosted many food vendors, who are largely similar in terms of food offerings and prices. However, interviews with customers and vendors indicated several characteristics that vendors use to distinguish themselves and attract customers. These include hygiene and food safety (i.e. having a clean table and clothes) and offering credit. In the words of one male snack vendor (age unknown), ‘To make sure that clients come back to me, I don’t make them pay in cash for the food they want if they don’t have money on them. Some people never forget those who help them in difficult times.’ Many interviewed miners confirmed choosing vendors based on ability to buy on credit.

Advertising and promotions for specific brands (e.g. posters, painted walls, billboards and shelf displays) are almost entirely absent from these markets, aside from branded labels on commercially processed foods. However, individual vendors confirmed that they offered incentives, such as gifts (e.g. free water), to attract or retain clients in the competitive market. Market observations showed some vendors using megaphones or speakers to advertise items.

### Personal food environment

Only 16·2 % of mining households have access to food from gardens, 59·9 % from agricultural production (within the prior two years), 47 % from poultry and 38·5 % from other livestock; 25 % have no access to food from own production (see Table 3). For those who do, production is fairly minor: only 2·5 % of respondents see agriculture as a main economic activity. This varies by site type, with those in temporary camps least likely to have access to food from own production and those in villages most likely to (e.g. 6·6 % of those in camps had farmed in the prior two years, *v*. 63·7 % in villages near towns); this trend aligns to variations in food availability in open-air markets. Table 6 summarises sources for main staples and key nutrient-dense foods from survey data, showing what percentage of households obtain half or more of each food from home production and confirming that households obtain limited food from own production and are largely dependent on markets. Water access is also limited among the sample, with slightly less than half having access to an improved water source. Nearly all households use informal open-air kitchens as their cooking facilities, with wood or charcoal as fuel.

**Table 6:** Main foods consumed and consumption from home production*

	Percentage of households (*n* 303)		
	Consuming never or rarely	Consuming every day or nearly everyday	Obtaining half or more of their consumption from own production
	%	%	%
Grains and staples			
Rice	3·0	96·0	42·2
Bread	11·6	62·0	5·5
Maize	42·2	27·7	41·4
Cassava	54·5	14·9	16·8
Pasta	56·4	12·0	2·0
Sweet potato	58·4	9·6	12·6
Vegetables			
Onion	5·0	91·1	15·6
Dark-green leafy vegetables	40·9	21·5	11·2
Eggplant	21·1	53·1	10·6
Tomato	39·9	24·8	9·4
Cabbage/lettuce	68·0	11·6	2·5
Carrot	92·7	0·0	4·6
Other vegetables	47·9	23·1	18·4
Fruits			
Mango	63·7	5·9	33·3
Papaya	89·8	1·0	10·9
Other fruit	61·7	5·3	6·6
Animal-source foods			
Fresh fish	21·0	64·7	2·5
Dried fish	54·8	21·5	0·9
Dairy products	47·5	18·8	5·3
Egg	42·2	15·2	2·0
Poultry	42·2	11·9	4·2
Meat	32·7	11·6	3·2
Nuts, seeds and legumes			
Peanut	16·2	55·4	24·3
Beans/cowpea	58·1	11·2	7·3
Lentils, nuts and seeds	92·7	0·7	3·3

* The sample size is 303 as these questions were asked in only wave 1, not both survey rounds. Within each food group, foods are sorted based on percentage of households consuming every day or nearly every day.

Understanding affordability requires relating prices to purchasing power. Interviews indicated that elevated prices exist alongside (somewhat) elevated incomes: ‘One earns money easily here,’ one 20-year-old female vendor explained, ‘but one spends it easily, too, due to the high prices.’ As explored in more detail elsewhere^(73)^, ASM miners are dependent on highly variable incomes, which depend on how much gold they find. Put simply, ‘if you find enough gold, you eat well’ (25-year-old single male miner). Indeed, qualitative data show considerable variation in food choice depending on daily income. One in-depth interview question asked respondents what food they would purchase on a hypothetical day when they made a given amount of money (the median reported revenue), then what they would buy if they had 50 % more or 50 % less. In general, increased income resulted in adding (or increasing) fish, meat or chicken; shifting from fish to meat; adding vegetables; increasing the amount of oil and having more sauce for a given amount of rice. A decrease resulted in no fish (or meat), less rice, no or different vegetables and using red palm oil instead of commercially processed vegetable oil. Affordability is clearly a key, but variable, constraint on food choices; participants’ responses suggested it was the main reason why certain foods are widely available but not commonly consumed (e.g. egg, meat, dairy). From the vendor perspective, affordability is essential for remaining competitive: ‘I set my prices so that (the miners) buy my food quickly, because if it is cheaper, you’ll have lots of clients,’ explained one 55-year-old female food vendor.

In addition to affordability, convenience is an important attribute of the personal food environment. At the site itself, interviewees opined that convenience manifests primarily in vendor location (i.e. their proximity to the mining shaft). ‘When I want something (to eat while mining),’ explained an 18-year-old single male miner, ‘I just go to whatever cafe is closest.’ Time places an additional constraint on food access and further emphasises convenience, as miners are working at the site from early morning until late afternoon. These schedules can lead to changes in mealtimes (e.g. about 20 % of respondents reported eating the morning meal earlier than normal and/or the evening meal later) or meal skipping. Given local gender norms, men who live without a female relative are largely dependent on food vendors for meals.

Considering desirability, taste, clean or hygienic preparation, family preferences, healthiness and price were cited by survey respondents as the most important characteristics sought in food, whereas it was seen as least important for food to be novel, modern or have attractive packaging. There were, however, some who saw packaging as a mark of food quality or cleanliness. Vendors share similar views on client preferences. ‘My only strategy for attracting clients,’ stated one 38-year-old female mobile snack vendor, ‘is to make my cakes delicious. People like what is delicious.’ Interviews also indicated that mining directly influences food preferences through a preference for foods and drinks seen to give energy – such as instant coffee or canned energy drinks: ‘Here is a mine,’ one 30-year-old male café operator explained, ‘so even before eating (whatever they eat), the miners will have a black coffee.’

The survey data ranking food characteristics suggested ‘health’ was an important factor in food choice; while this might be assumed to refer to nutritional content, qualitative interviews suggest that understanding of nutrition is very limited. Indeed, many see ‘healthy’ food as being the same as ‘safe or hygienic food’; when asked to cite healthy foods, a greater percentage named juice drinks or soft drinks (29 %) than named fruit (19 %) or vegetables (17 %).

## Discussion

**Fig. 1:**
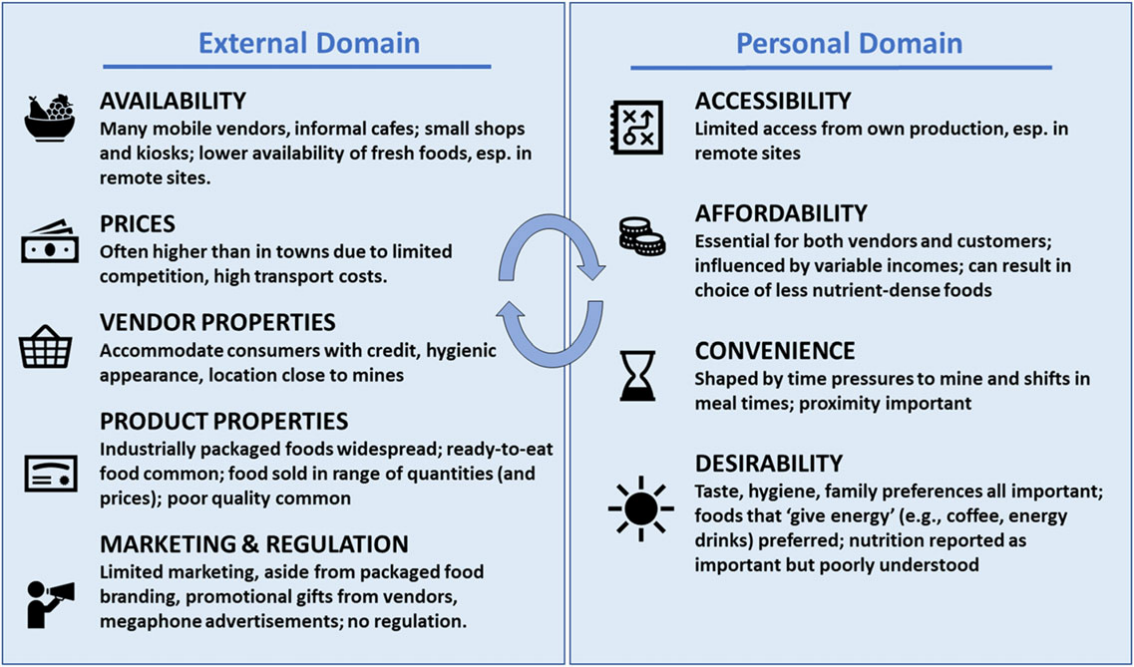
Summary of Main Results, by Food Environment Characteristic

Using diverse methods, this paper has analysed the personal and external food environments of artisanal mining communities in Guinea. Figure 1 summarises the key characteristics uncovered.

From examining this figure, it is clear that this rural setting has many characteristics typically associated with urban areas. Packaged and prepared foods are widely available, including commercially processed drinks and snacks. Consumers are largely dependent on cash incomes, with limited access to food from own production. They place a premium on convenience and rapidly accessible food. However, they also have some agricultural production and limited access to options for purchasing non-prepared foods (e.g. no supermarkets, in some cases, no daily open-air market), refrigeration and cooking technology and complementary services (e.g. piped water) and have physically demanding livelihoods dependent on natural resources. These communities’ food environments thus mix characteristics traditionally associated with rural or urban populations^(24)^. While these sites are unusual, their characteristics are by no means unique. Even in rural areas of sub-Saharan Africa, households purchase much of their food^(18,19)^. Consumption of commercially processed foods is fairly common in rural areas – even in West Africa, even by young children^(74)^. This complexity underlines the importance of further interrogating food environments across diverse LMIC settings, including rural areas.

Food environments alone do not determine food choices, and research in high-income settings has suggested personal perceptions may be stronger determinants than environmental characteristics, such as prices and availability^(75)^. However, there are some clear ways in which these environments’ characteristics may influence diets and nutrition. Limited access to own-produced foods, along with low, variable cash incomes, creates a situation that fosters food insecurity and diets low in diversity – despite widespread availability of nutrient-dense foods, such as meat and eggs, in many markets. This disconnect between food availability and food/nutrition security emphasises the need for more research on food affordability in LMIC^(67,76–78)^: availability means little when not accompanied by affordability. However, the literature generally considers food affordability to be a largely static concept, comparing food prices to average annual/monthly incomes or expenditures, without considering the substantial income variability that affects the miners studied here – and many other informal workers. For affordability metrics to give an accurate picture of the constraints consumers face, income variability must be given more primacy. In this setting, income variability is likely to be an even more important driver for women, as their roles in mining (hauling and washing) are less lucrative than those of men (digging and operating machines)^(73)^, underlining how the personal food environment may vary by socio-demographic group.

This study also uncovered easy availability of affordable, convenient and nutrient-poor commercially processed foods. This availability arises at mining sites due to vendors recognising potential demand, which intersects with miners’ desire to have more energy to mine and to consume ‘clean’ foods with lower risk of contamination in an unclean environment. Jointly, these factors lead to surprisingly frequent consumption of commercially processed sweets and energy drinks, including by young children. Such consumption (especially in a setting of overall food insecurity) by young children could lead to nutrient displacement^(79)^; in adults, the consumption of ultra-processed foods (a subcategory of commercially processed foods) has been connected with obesity and non-communicable diseases^(80,81)^. However, their consumption is rarely seen as a major issue in rural areas of lower-income countries, on the understanding that such areas have not yet ‘transitioned’ to modern food systems^(17)^. This study underlines the importance of broadening that lens and considering availability of commercially processed foods in rural food environments.

In the more remote camps, moreover, the availability of affordable, fresh, nutritious products was particularly low. Food diversity in rural markets has been shown to influence children’s dietary diversity^(21)^; this is likely even more true in mining camps, where there are few options for home food production. Indeed, many dietary indicators were found to be poorest in the more remote mining camps.

The results presented here also show how food environments are dynamic and emerge from an interaction of suppliers and consumers amid larger contextual forces. In this setting, the widespread availability of ready-made and commercially processed food, as well as limited access to food from own production, is by-products of the predominant local livelihood. As compared with an agricultural livelihood, ASM offers the potential for greater incomes^(26,27)^ but also more day-to-day variability in income^(73)^. While ASM can complement agriculture^(26,51,82–85)^, it tends to reduce reliance on farming; for migrants, it can mean limited access to livestock or land to produce crops. ASM is a high-pressure, competitive and time-consuming occupation, leading miners to place greater weight on convenience and to seek foods they hope will give them energy. Prepared food vendors, once rare in such rural settings, emerge to cater to these needs. In response to customer demand, they offer prepared meals and energy drinks within steps of mine shafts. Their focus on hygiene (amid an unclean environment) and offering dishes at a range of prices and on credit (when facing clients with irregular income) further demonstrate the ways in which they respond to customers’ needs. The resultant food environment is thus a product of supply and demand interacting within a given context and a larger food system.

There are numerous limitations of this study. Mine locations change often, and there are challenges with accessing them. Different villages and markets were thus sampled for different parts of data collection, and it was not possible to create a truly random or population-representative sample. Data were collected only in mining areas and only over an 18-month period, making it impossible to compare trends over time or to non-mining areas. The study’s strengths include an in-depth, comprehensive approach that used diverse methods over an extended period of time. The result is a detailed picture of the personal and external food environments within a rural, non-agricultural LMIC setting.

The results have implications for policy and programming to improve nutrition in similar areas. In this setting, food availability is not a major barrier to consumption, whereas affordability is largely due to highly variable incomes. As such, efforts could be made to stabilise incomes (e.g. through savings groups or mobile banking). Formalisation of property rights could also have a positive effect, as it stabilises income from mining and allows for more advance budgeting and planning^(86)^. Encouraging agriculture to improve food accessibility is unlikely to be attractive to those engaged in mining, given the appeal of earning a fast cash income and the time-demanding nature of mining. However, time-efficient, low-labour options for food production, such as keyhole gardens or chicken-rearing, could be tested. Vendors could be supported with training to offer more nutrient-dense options and fewer ultra-processed foods – such as by greater use of relatively affordable nutrient-dense foods (e.g. organ meats, dried fish). To reshape food desirability, messaging could work to counteract miners’ common belief that nutrient-poor ultra-processed foods/beverages (e.g. energy drinks) are good sources of energy for mining. Finally, while government regulation of food quality and safety at sites is effectively non-existent and infeasible to expect in the short term, given limited resources, site infrastructure could be improved to support food safety. When translated into such tailored intervention approaches, greater attention to non-agricultural rural food environments in research and policy could help reduce malnutrition among some of Africa’s most vulnerable communities.
